# Sample selection, recruitment and participation rates in health examination surveys in Europe – experience from seven national surveys

**DOI:** 10.1186/s12874-015-0072-4

**Published:** 2015-10-05

**Authors:** Jennifer S. Mindell, Simona Giampaoli, Antje Goesswald, Panagiotis Kamtsiuris, Charlotte Mann, Satu Männistö, Karen Morgan, Nicola J. Shelton, WM Monique Verschuren, Hanna Tolonen, on behalf of the HES Response Rate Group

**Affiliations:** Research Department of Epidemiology & Public Health, UCL, 1-19 Torrington Place, London, WC1E 6BT UK; Istituto Superiore di Sanità, Viale Regina Elena n. 299, Rome, Italy; Department 2 Epidemiology and Health Monitoring Division 25, Robert Koch Institute, Examination surveys and Cohort studies, General-Pape-Str. 62-66, Berlin, 12101 Germany; Department of Health, National Institute for Health and Welfare (THL), P.O. Box 30, Helsinki, FI-00271 Finland; Department of Psychology, Royal College of Surgeons in Ireland, Dublin, Ireland; Perdana University, Serdang, Malaysia; Department Chronic Diseases Determinants, Center for Nutrition, Prevention and Health Services, National Institute for Public Health and the Environment, P.O. Box 1, Bilthoven, 3720 BA The Netherlands

**Keywords:** Europe, Health examination survey, Sampling frame, Sample size, Eligibility, Recruitment, Incentives, Participation rates, Response

## Abstract

**Background:**

Health examination surveys (HESs), carried out in Europe since the 1950’s, provide valuable information about the general population’s health for health monitoring, policy making, and research. Survey participation rates, important for representativeness, have been falling. International comparisons are hampered by differing exclusion criteria and definitions for non-response.

**Method:**

Information was collected about seven national HESs in Europe conducted in 2007–2012. These surveys can be classified into household and individual-based surveys, depending on the sampling frames used. Participation rates of randomly selected adult samples were calculated for four survey modules using standardised definitions and compared by sex, age-group, geographical areas within countries, and over time, where possible.

**Results:**

All surveys covered residents not just citizens; three countries excluded those in institutions. In two surveys, physical examinations and blood sample collection were conducted at the participants’ home; the others occurred at examination clinics. Recruitment processes varied considerably between surveys. Monetary incentives were used in four surveys. Initial participation rates aged 35–64 were 45 % in the Netherlands (phase II), 54 % in Germany (new and previous participants combined), 55 % in Italy, and 65 % in Finland. In Ireland, England and Scotland, household participation rates were 66 %, 66 % and 63 % respectively. Participation rates were generally higher in women and increased with age. Almost all participants attending an examination centre agreed to all modules but surveys conducted in the participants’ home had falling responses to each stage. Participation rates in most primate cities were substantially lower than the national average. Age-standardized response rates to blood pressure measurement among those aged 35–64 in Finland, Germany and England fell by 0.7-1.5 percentage points p.a. between 1998–2002 and 2010–2012. Longer trends in some countries show a more marked fall.

**Conclusions:**

The coverage of the general population in these seven national HESs was good, based on the sampling frames used and the sample sizes. Pre-notification and reminders were used effectively in those with highest participation rates. Participation rates varied by age, sex, geographical area, and survey design. They have fallen in most countries; the Netherlands data shows that they can be maintained at higher levels but at much higher cost.

## Background

Sample based health examination surveys have been conducted in Europe since the late 1950’s and in the USA since 1962 [1]. In several WHO member countries, health examination surveys are conducted under STEPS framework [2]. These surveys have provided valuable, objective information about the health of the general population, which can be used as a basis of health monitoring [3] and health policy making [4] as well as for evaluation of health promotion activities and research.

The best value for health examination surveys is obtained when results are reliable and represent the general population well. Hospital-based surveys or other surveys targeting only on patients are limited to those receiving healthcare. They will not identify undiagnosed disease, such as cases of hypertension, or diabetes [5]. Additionally, standardization of measurement protocols can be better controlled in specially designed health examination surveys than when data is collected from existing health care systems [6]. Standardized health examination surveys can also provide data which is comparable across populations.

The representativeness of the health examination survey data is strongly dependent on sample selection procedures and on the participation rates achieved, and also specification, measurement and processing errors’ effect on representativeness. In questionnaire surveys, the mode of data collection may also affect participation rate [7]. The quality of different sampling frames available varies considerably both within and between countries [1]. Even when the best available sampling frames are used and probability samples are selected to obtain the best representation and coverage of the target population, non-participation in the survey may cause uncertainty regarding the representativeness of the survey results.

In the 1980s, it was possible to obtain a participation rate of 80 % in health examination surveys. Nowadays, participation rates of 40-50 % are common [1, 8] and this is becoming a major problem. Non-participation is selective, i.e. non-participants are more often young men, single and from lower socio-economic groups, having worse self-reported health, and they are more likely to be smokers than survey participants [9–14]. Survey non-participants also have higher total and cause specific mortality than survey participants [15–17].

In 1984–85, a response rate of 73.5 % was considered normal [18]. Decreasing survey participation rates have occurred in the USA [19] and Europe [20–24]. For example, in Finland, response rates for a health interview survey fell from 84 % in 1978 to 59 % in 2002 in men and from 85 % to 71 % in women aged 25–64; the rate of decline varied considerably by age and sex [23].

Much research has been done on the effects of different recruitment methods on survey participation in questionnaire based surveys. A Cochrane review evaluated 47 studies (from 1954 to 2008) on the effect of pre-notification before sending the actual questionnaire for survey invitees. Mailed pre-notification increased participation in the surveys, while telephone pre-notification did not have any significant effect [25]. Previous studies have shown that monetary incentives increase participation rates, particularly if provided unconditional on participation [25, 26].

Only a few studies have investigated the effect of recruitment methods on health examination surveys [27–29]. It could be expected that most of the findings from questionnaire based surveys would be valid for health examination surveys also. It is important to examine the effects of different recruitment methods on survey participation. We need to find the most cost-effective way to obtain as high participation rates as possible.

The reliability and generalizability of survey results depend on the data collected being representative of the population the survey is intending to describe. Sampling methods, non-response, and measurement errors are the main issues that affect survey error. While random probability sampling from an appropriate sampling frame and robust measurement protocols with adequate training and quality assurance are within the researcher’s control, non-response is more dependent on the sampled population and is of particular concern, [14] although it can be influenced by recruitment and promotion strategies.

A 2007 review discussed decreasing participation in epidemiological surveys [30]. Explanations suggested included the increasing number of research studies plus the proliferation of political polls, telephone marketing, and marketing surveys that may resemble scientific surveys. Survey information that arrives in the post or by telephone may be assumed to be “junk”, arriving together with unsolicited mail or calls from commercial sources, building additional barriers to considering participation when contacted.

Health examination surveys (HESs) sampling the general population nationally have been conducted recently and on a number of previous occasions in several European countries and are being encouraged more widely [6]. One problem of international response rate comparisons is the use of different definitions of non-response.

The aim of this paper is to describe and compare sampling and recruitment methods used in national health examination surveys in seven European countries. We evaluate the representativeness of the survey for the general population; participation rates, analysed using standardised definitions, for various measurements by population sub-groups; and changes in these over time; and the different participation rates achieved.

## Methods

### Data

Survey organizers from seven European countries which had conducted at least two national health examination surveys (HESs) provided information about sampling and recruitment and details of participation rates for questionnaire or interview, specific measurements, and providing a blood sample, using standardised definitions.

### Individual-based surveys

**Fig. 1:**
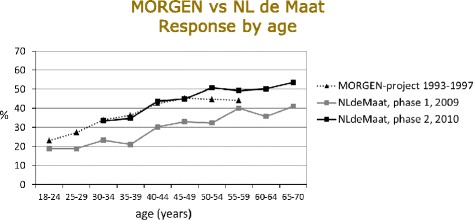
Changes in age-specific participation rates, The Netherlands, 1993–97 to 2010

Four countries ran individual-based surveys. In Finland, population-based health surveys have been conducted at five-year intervals since 1972, initially in Eastern Finland only. In 1982–1992, the surveys were part of the multinational WHO MONICA Project [31]; since 1992, the surveys have been called *The National FINRISK Study*, covering five areas in 2012 [3] Germany has organised national health interview and examination surveys since 1984. The *Studie zur Gesundheit Erwachsener in Deutschland* (DEGS) *[German Health Interview and Examination Survey for Adults]* was conducted from November 2008 to December 2011, including both a follow-up of the 1998 survey participants and a new sample [32, 33]. In Italy, the first national health examination survey (OEC) was conducted 1998–2002 and the second survey, Observatorio Epidemiologico Cardiovascolare/Health Examination Survey (*OEC/HES*) in 2008-2012 [34]. In the Netherlands, large-scale population-based health surveys were conducted annually 1987–1997. The most recent, *Nederland de Maat Genomen (NLdeMaat) (Measuring the Netherlands)*, was carried out in two phases: Phase I in 2009 and Phase II in 2010 [35, 36]. The response rate in phase I in 2009 was only 30 % for those aged 18y and over (15 % aged 18-29y) (Fig. 1). Several changes were made, including setting the minimum age, increase of the token of appreciation, and changes in the invitation procedures, and the examination hours offered. The results from the Netherlands presented in this paper are predominantly from phase II.

### Household-based surveys

Three countries held household-based surveys. The *Health Survey for England* (HSE), has been conducted on a new sample annually since 1991 [37, 38]. All adults (maximum 10) are eligible for inclusion. *Scottish Health Surveys* (SHeS) were conducted in 1995 and 1998, when only one adult per household was sampled, and in 2003 then annually from 2008 onwards, with all adults (maximum 10) included [39, 40]. For each of these, an interviewer visits the home to collect information and measure height and weight. In Scotland (up to SHeS2011) and England, a nurse visits subsequently to take further measurements (including blood pressure) and collect biological samples, including a blood sample in most years.

In the most recent *Survey of Lifestyle And Nutrition* (SLÁN) in Ireland in 2007, one adult per household was randomly selected for a health interview [41]. A sub-sample of those aged under 45 years also had height, weight and waist circumference measured, while a second sub-sample of those aged over 45 years were invited to have a full health examination.

Each survey received research ethics approval from a relevant committee or institution prior to fieldwork: *FINRISK 2012:* Helsingin ja Uudenmaan sairaanhoitopiiri, Koordinoiva eettinen toimikunta, Helsinki (162/13/03/00/2011); *DEGS 2008–2011:* Ethik Kommission, Charité, Universitätsmedizin, Berlin (EA2/047/08); *SLAN 2007:* Royal College of Surgeons of Ireland, Dublin (RCSI REC 204 & 206); *OEC/HES 2008–2012:* Ethical Committee of the Istituto Superiore di Sanità, Rome (CE-ISS 08/208 and CE-ISS09-278), plus each relevant local ethics committee; *NldeMaat 2009–2010:* Medisch Ethische Toetsingscommissie, Universitair Medisch Centrum, Utrecht (08–420); *HSE 2011:* Oxfordshire REC A, National Research Ethics Service, NHS, Oxford (10/H0604/56); and *SHeS 2011:* Research Committee for Wales, Cardiff (08/MRE02/62). Each survey participant had provided informed consent.

### Analysis

Published national reports varied in how participation rates were calculated, including treatment of those who were away. Some used refusal rates; others reported broader non-response, including not contacted; contacted but not eligible (e.g. language difficulties, lack of mental capacity); and refusals. For this study, functional equivalence for participation rates was obtained as follows:

- Numerators were defined as those interviewed; or those consenting to have their weight/blood pressure measured, or provide a blood sample. Technical problems with obtaining the measurement or sample and factors that would exclude these from analysis within the survey (e.g. having smoked too recently before blood pressure was measured, or the blood sample being lost) were ignored: the aim was to assess differences in willingness to participate.
- For interview participation rates, the denominator was defined as all those sampled, except where the sampling frame was wrong (the address not existing or not being a private residence, for household-based surveys where the fieldstaff visited the address); the invitation letter was returned as undeliverable or ‘not known at that address’; or people died before their clinic appointment (for individual-based surveys).
- For participation (consent) rates for physical and biological measurements, those capable of giving consent but ineligible for that specific measurement were excluded from the denominator (e.g. the weight of bed-bound individuals; pregnant women were not measured in England and Scotland). Other ineligible or excluded individuals, such as those who were away, in hospital or unable to give informed consent due to language problems or learning disability, remained in the denominator.

For household surveys sampling by address, in which more than one adult per household was selected (England and Scotland), interview participation rates were calculated both restricted to co-operating households (where at least one adult responded, providing household information as well as individual data), and overall, assuming the household composition of non-responding households was the same as co-operating households. For Ireland, the sampling frame for the examination survey was a sub-sample of those aged 45 years or older who were interviewed in the household survey, so participation rates have been multiplied by the household participation rate.

The four participation rate outcomes were: consent rate for blood pressure measurement (primary outcome measure), participation in the interview, and consent for weight measurement and for providing a blood sample. These represented four stages of the surveys. Using the definitions above, we determined participation rates by age and sex, geography, and year. Depending on the data available, national participation rates were compared with data from the capital city (Greater London, Rome), the primate city^1^ (Glasgow City/Greater Glasgow area), [42] or major metropolitan areas (Berlin, Munich, Hamburg and Frankfurt combined, Helsinki-Vantaa).

Where possible, age-standardized rates using the European Standard Population [43] were calculated for participants aged 35–64 years, covering the age-groups included in most surveys. Due to differences in the age-groups included and the calculations of participation rates between individual-based and household-based surveys, results presented are not directly comparable between countries.

## Results

### Sampling in European national health examination surveys

Sampling frames in these seven national HESs can be divided into two groups; individual-based and household-based. All individual-based sampling frames were population registers, either national or local. Population registers, commonly available in Europe, list people living in the country/region but obviously there are some technical problems with homeless individuals, etc. They may be listed in the population register but without a permanent address. In three surveys, household-based -sampling frames were used because of the lack of population registers in those countries: in these surveys, the sampling frames were address files (Table 1).

**Table 1:** Sampling in seven national health examination surveys in Europe

Survey	Year(s) of the survey	Sampling frame	Target population	Sample selection	Original sample size	Eligible sample size	% of sample ineligible	Ineligibility criteria
England – Health Survey for England	2011	Address file – the small user Postcode Address File	People of all ages living in private households.	1. Random sample of postcode sectors, stratified by region and % non-manual occupation.	8 992 households	8 088 households	10 %	Business or institutions, vacant buildings, demolished buildings, building still being built.
				2. Random sample of private addresses within selected postcode sectors from the Postcode Address File.				
				3. Up to 10 adults and two children in each selected household.				
Finland – FINRISK Study	2012	Population register	People aged 25–74 years living in five regions of Finland.	A random sample of individuals stratified by sex, 10-year age group and five regions.	10 000 individuals	9 905 individuals	1 %	Died, moved away from research area.
Germany – DEGS	2008-2011	Local population registers	People aged 18–79 years and living in Germany.	Participants of former survey the German National Health Interview and Examination Survey 1998 (GNHIES98) plus new random sample:	17 117 individuals	15 974 individuals	7 %	For new sample:
								Died, moved away from research area.
								Unable to understand basic German.
				1. Random sample of points within Germany.				
								For re-invited participants from GNHIES98:
				2. Random sample of individuals within these points.				
								Died or moved abroad.
Ireland – SLAN	2007	Address file – the GeoDirectory	People aged 18 years and over living in private households.	1. Random sample of sampling points based on aggregates of townlands.	19 185 households for interview	16 681 households for interview	13 %	Vacant buildings, non-residential building, demolished building, address which could not be located.
			18–44 years old had questionnaire and anthropometric measurements at home. 45+ years old were invited to the more extensive examination at the examination centre.					
				2. Systematic sample of addresses within selected sampling points.				
				3. A selection of a person within household by simple randomization procedure by next birthday rule.				
Italy – OEC/HES	2008-2012	Population register	People aged 35–79 years, living in the 20 Italian Regions.	Screening centres selected in each region based on availability of personnel, space, laboratory facilities and willingness to collaborate on study. Within each selected municipality of screening centre, a random sample of participants was selected by age-group and sex.	17 052 individuals	16 447 individuals	4 %	Undelivered letter, died, emigrates, working outside the residence area for all survey period.
Netherlands – NLdeMaat	2009-2010	Population register	People aged 18–70 years (phase I) and 30–70 years (phase II) living in five Dutch towns.	1. Division of country into five regions.	15 000 individuals	14 163 individuals	6 %	Diet, moved away from the region.
				2. Random sample of three sampling points (towns) from each region.				
				3. Random sample individuals stratified by sex and 10-year age group.				
Scotland – Scottish Health Survey	2010	Address file – the small user Postcode Address File	People of all ages living in private households.	1. Random sample of postcode sectors, stratified by area and deprivation.	8 382 households (2 194 households for nurse visit)	7 564 households	10 %	Business or institutions, vacant buildings, demolished buildings, building still being built.
				2. Random sample of private addresses within selected postcode sectors.				
				3. All adults and up to two children in each selected household.				

All surveys covered both men and women. In England and Scotland, all people (without age limit) were included in the survey. In Ireland, people from age of 18 years without an upper age limit were included; those aged 18–44 years had their height, weight and waist circumference measured and those 45+ years old were invited for a full physical examination. The other four surveys had narrower age limits. The age group 35–64 years was common to all seven surveys (Table 1).

In each survey, all people living in the country were included in the target population to the extent it was possible to list them in the sampling frames, i.e. the target population was residents not citizens. For practical reasons, in the three household surveys, only people living in private households were included, i.e. institutionalized people were excluded from the target population (Table 1).

In each survey, probability sampling was used. In three surveys (Finland, Italy and the Netherlands), survey areas were selected based on availability/feasibility criteria and individuals within each selected areas were selected using random sampling from the population register. In these surveys, samples were stratified by age group and sex. In Germany, where part of the sample was based on re-invitation of participants of the previous HES (conducted in 1998), the additional new sample was selected using a two stage random sample [33]. In the three surveys using household sampling, two stage sampling was used. In the first stage, the local areas were selected (postcode sectors). In the second stage, a set number of households were selected from each of the selected areas. Within selected households, sampling procedures to select individuals varied between surveys (Table 1).

The original sample size in these surveys varied from 10,000 to 17,117 individuals in surveys based on samples of individuals and from 8,736 to 19,185 households in surveys based on household sampling (Table 1).

In individual-based surveys, people who had died or moved out from the survey area were considered as ineligible for the survey. In Germany, people who did not understand basic German and in Italy people who were outside the survey area for the entire survey period were also classified as ineligible for the survey. In household-based surveys, vacant, demolished, and non-residential buildings were classified as ineligible (Table 1).

Eligible sample sizes (original sample size minus those not eligible) for individual based surveys varied from 9,905 to 16,477 individuals. For household surveys, eligible sample sizes were from 7,564 to 16,681 households. In surveys using population registers as sampling frames, the proportion who were ineligible was 1 %-7 % and in surveys using address lists as sampling frames, 10 %-13 % (Table 1).

### Recruitment

In all surveys except in Finland and Ireland, the first contact with survey invitees was either an advance letter or an invitation letter. In Finland, survey invitees received first a short message service (SMS) text message which was then shortly followed by an invitation letter. In Ireland, the invitation to take part in the health examination was given by the interviewer who located the address (Table 2).

**Table 2:** Recruitment methods used in seven national health examination surveys in Europe

Survey	Form of invitation	Recruitment process	Incentives	Questionnaire administration	Examination place	Examination time (days of the week)	Examination time (time of the day)
England – HSE	Advance letter, notifying them an interviewer will call at their home, plus two information leaflets.	Interviewer visits a household to schedule an interview (min 6 attempts).	Unconditional £5 voucher per household, feedback on physical measurements and blood analysis results.	By interview during the separate visit before examination (nurse visit).	Participant’s home	Monday-Sunday	08:00–22:00
Finland – FINRISK Study	Pre-notification SMS message followed by invitation letter which includes proposed examination time with possibility to change the time.	A reminder SMS message day before given appointment time. If person did not show to the appointment, 1–2 phone calls. If no contact by phone, a reminder letter with questionnaire.	Feedback on physical measurements and blood analysis results.	Self-administered questionnaire sent with invitation letter. Questionnaire checked at the beginning of the examination visit.	Examination centre	Monday-Friday	11:00–18:00
Germany – DEGS	Invitation letter with a comprehensive information sheet, a reply card and questionnaire.	If no reply card received back, a reminder letter, after that phone calls and last home visits.	30 € cash per participant, feedback on physical measurements and blood analysis results.	Self-administered questionnaire sent with invitation letter. Questionnaire checked at the beginning of the examination visit.	Examination centre	Tuesday-Wednesday	12:30–22:00
						Thursday-Friday	7:30–17:00
						Saturday	7:30–15:00
				Second self-administrated questionnaire and interviews at the examination site.			
Ireland – SLAN	Face-to-face invitation from interviewer who located the address.	Home visit as part of health interview survey.	Full report from medical staff on physical measurements and blood/urine analysis results.	By interview at the home of the participant before examination and for those participating in full HES part, additional questionnaire asked by a nurse at the examination centre/home visit.	Examination centre or home	Monday-Sunday	All day
Italy – OEC/HES	Invitation letter which included proposed appointment and contact details for more information or to change the appointment time.	If person neither attended the given appointment time nor changed that, phone calls. If no contact by phone, a reminder letter with new appointment time. The last attempt was a personal phone call.	Feedback on physical measurements (including bone densitometry, spirometry and ECG) and blood analysis results, and lifestyle advice. No financial incentive.	By interview at the examination centre	Examination centre	Monday-Friday	08:00–17:00
							Later on request
						Sometimes on Saturday and Sunday	
Netherlands – NLdeMaat	Phase I: Invitation with a return card to make an appointment.	Phase I: If no reply card within 2 weeks, a reminder letter. If no reply to reminder in 2 weeks, a phone call (max 6 attempts).	Phase I: 10€ voucher per participant, feedback on physical measurements and blood analysis results.	Self-administered. Questionnaire checked at the beginning of the examination visit.	Examination centre	Phase I: Monday-Friday, Saturday	Phase I: Mon-Fri: 07:00–11:00
							Sat: 09:00–14:00
	Phase II: Invitation with an appointment card.	Phase II: If no reply card returned in 2 weeks, a home visit to recruit (max 6 attempts).	Phase II: 50€ voucher per participant, feedback on physical measurements and blood sample analysis results.	As for phase I	As for phase I	Phase II: Monday-Friday, Saturday	Phase II: Mon-Fri: 07:00–11:00, 15:00–20:00
							Sat: 09:00–14:00
Scotland – Scottish Health Survey	Advance letter, notifying them an interviewer will call at their home, plus information leaflet.	Interviewer visits a household to schedule an interview (min 6 attempts).	Unconditional £5 voucher per household, feedback on physical measurements and blood analysis results.	By interview during the separate visit before examination (nurse visit).	Participant’s home	Monday-Sunday	08:00–22:00

*BP* Blood pressure

The recruitment processes after the initial contact with the survey invitees varied between countries. There were different combinations of reminder letters, phone calls and home visits. The number of contact attempts varied from one upwards (Table 2).

In all surveys, feedback about the examination results was provided for survey participants. In Italy lifestyle advice was also provided for survey participants. In England, Germany, the Netherlands and Scotland, monetary incentives were used to promote participation. In Germany, cash was provided at the end of the health examination, while in other surveys, vouchers were given. In the Netherlands, receiving the voucher was conditional on participation while in England and Scotland, the voucher was sent to the household with the advance letter, i.e. it was unconditional on participation (Table 2).

In England, Ireland and Scotland, face-to-face interviews and self-completed questionnaires were filled in during the interview at the survey participant’s home. In Italy, an interview was conducted at the examination centre. In Finland, Germany and the Netherlands, questionnaires were sent to the survey invitees and they were filled in before coming to the examination site. The questionnaires were checked by survey staff during the examination visit. In Germany, additional information was collected by a second self-administered questionnaire and personal interviews at the examination site (Table 2).

In England and Scotland, health examinations were carried out by trained nurses in the survey participant’s home; additional questions were asked by the nurse during this visit. In other countries, health examinations were conducted in fixed examination centres (Table 2).

In Finland, health examinations were carried out during working days (Monday-Friday) while in other countries examination times were also offered at the weekends (Saturday and/or Sunday) (Table 2).

The times of the day when health examinations were carried out also varied between countries. In England and Scotland, which conducted the health examinations at the survey participant’s home, times throughout the day from morning to late evening were offered. In Ireland, appointments were available all day. The time frame for the examinations was narrowest in Finland: from 11:00 until 18:00. In Germany and Italy both morning and evening hours were available. In the Netherlands, the survey started (phase I) with only morning hours but to boost participation, evening hours were offered in phase II of the survey (Table 2).

### Participation rates

The common age group in all surveys was 35–64 years, except in Ireland with no blood pressure measurements or sample collection for participants aged 35–44. Participation rates were generally higher in women than in men, except in Ireland, where they were equal, and in Germany, where men aged 35–64 had a higher participation rate. Participation rates increased with age up to 64 in all surveys. For older age groups, participation rate was lower in women in Germany and in both sexes in Italy (Table 3).

**Table 3:** Consent rates in recent HES by age and sex

Country	Survey^a^	Year	Number in sample		Interviewed (%)		Weight measured (%)		BP measured (%)		Blood sample taken (%)	
			Men	Women	Men	Women	Men	Women	Men	Women	Men	Women
Individual sampling frame												
Finland	FINRISK	2012										
25-34			983	980	45	61	41	53	41	53	41	53
35-44			994	989	55	65	50	59	50	59	50	59
45-54			992	995	61	70	56	63	56	63	56	63
55-64			991	997	67	73	61	66	61	66	61	66
65-74			988	996	77	73	72	67	72	66	72	67
35-64^b^			2980	2981	61	69	55	62	55	62	55	62
Germany^c^	DEGS	2008-2011										
25-34			431	501	36	43	36	38	36	38	32	34
35-44			583	681	44	53	43	44	43	44	37	44
45-54			775	899	52	60	50	49	50	49	42	50
55-64			684	756	56	58	54	49	54	49	45	46
65-74			816	843	58	55	56	51	56	51	48	43
35-64^b^			2042	2336	51	57	49	48	49	48	41	47
Italy	OEC/HES	2008-2012										
35-44			1967	1911	49	51	49	51	49	51	49	51
45-54			1836	1809	57	61	57	61	57	61	57	60
55-64			1807	1776	60	60	60	60	60	60	59	60
65-74			1773	1803	55	54	55	54	55	54	54	53
75-79			833	932	52	40	52	40	52	40	52	40
35-64^b^			5610	5496	55	57	55	57	55	57	55	57
Netherlands^d^	NLdeMaat Phase 2	2010										
35-44			135	171	35	43	35	43	35	43	35	43
45-54			186	228	44	52	44	52	44	52	44	52
55-64			163	167	49	50	49	50	49	50	49	50
35-64			484	566	42	48	42	48	42	48	42	48
Hybrid sampling												
Ireland^e f^	SLAN	2007										
45-54			177	271	41	41	41	41	41	41	39	40
55-64			149	205	41	41	41	41	41	41	40	39
65-74			151	149	41	41	41	41	41	41	39	39
45-64^e f^			326	476	41	41	41	41	41	41	39	39
Household sampling frame												
England^e^	HSE	2011										
25-34			696	642	79	88	68	71	47	54	35	45
35-44			826	825	82	95	70	79	53	64	41	56
45-54			823	876	81	93	70	78	52	63	41	55
55-64			721	886	87	94	75	79	63	69	50	59
65-74			561	822	90	96	78	81	66	68	48	59
35-64^b^			4643	5242	83	94	71	79	55	65	44	56
Scotland^e^	SHeS	2010										
25-34			392	534	72	90	64	72	36	46	27	36
35-44			443	637	76	94	67	77	42	48	37	40
45-54			530	721	78	94	68	80	43	57	39	48
55-64			514	655	83	94	71	80	51	56	46	46
65-74			454	534	92	96	80	80	51	54	38	44
35-64 ^b^			1487	2013	79	94	69	79	45	54	41	45

^a^FINRISK: National FINRISK Study; DEGS: German Health Interview and Examination Survey for Adults; OEC/HES: Osservatorio Epidemiologico Cardiovascolare/Health Examination Survey; NLdeMaat: Nederlands de Maat Genomen; SLAN: Survey of Lifestyle And Nutrition; HSE: Health Survey for England; SHeS: Scottish Health Survey

^b^Age-standardised

^c^Results show the combined response rate of the new and the reinvited sample of the previous survey [16]

^d^Results shows are for Phase II of NLdeMaat

^e^Based on co-operating households (i.e. at least one person was interviewed)

^f^Note different age groups: Ireland did not include age group 35–44 years for blood pressure measurements and sample collection

In surveys where all physical measurements and sample collection were done at examination centres, participation rates differed little between weight measurement, blood pressure measurement and blood sample collection, varying by only 0–3 percentage points (pp) in Italy, Ireland and the Netherlands; in Finland, blood sample consent rates were 5–8 pp lower than interview rates; in Germany, blood sample consent rates were 4–9 pp lower than the BP measurements. In Germany, some of the difference was due to people who were unwilling or unable to attend the centre having a telephone interview.

However, in England and Scotland, the fully household-based surveys where two visits by survey staff were required and measurements were conducted in the participants’ home, there was a marked reduction in participation rate for each successive measure in the survey of 38–46 pp among adults in participating households (Table 3).

Except for Finland, participation rates for capital or primate cities and major metropolitan areas were consistently around 8–15 pp lower than the national average (Table 4). In England and Scotland, differences occurred in both the initial household participation rate and the proportion having a nurse visit but individual participation rates within co-operating households varied little from the national average.

**Table 4:** Participation rates for different outcomes, for national and primate cities/metropolitan areas^a^ ^b^

Country	Year	Age range	Household response rate	Number in sample		Interviewed (%)		Weight measured (%)		BP measured (%)		Blood sample taken (%)	
				Men	Women	Men	Women	Men	Women	Men	Women	Men	Women
Individual sampling frame													
Finland	2012	25-74											
Helsinki- Vantaa				986	990	61	63	55	58	55	58	55	58
National survey				4948	4957	61	68	56	62	56	61	56	61
Germany	2008 to 2011												
Metropolitan areas^c^		25-74		242	300	41	46	41	46	41	46	33	36
National Survey				3289	3683	50	54	48	47	48	47	41	44
Italy	2008to 2012	35-79											
Rome				762	805	41	39	41	39	41	39	41	39
National Survey				8216	8231	55	55	55	55	55	55	54	54
Household sampling frame													
England^d^	2011	16+											
Greater London			56	538	627	81	88	68	72	44	52	35	44
National Survey			66	4643	5242	82	91	70	75	54	60	44	51
Scotland^d^	2010	16+											
Glasgow City^e^			51	313	406	79	89	68	73	30	38	25	30
Greater Glasgow & Clyde Health Board			55	582	840	76	90	62	68	31	41	26	34
National Survey			63	2896	3874	78	92	68	75	43	50	36	41

^a^Information on response rate by geographical area was not available by age-group in household-based surveys, thus all ages of the adult samples were included for this table. Therefore the age-ranges covered by each survey in this table varies. Comparisons should be within (not between) countries

^b^No data available for Ireland or The Netherlands

^c^Berlin, Munich, Cologne, and Hamburg

^d^Among co-operating households (i.e. at least one adult was interviewed), apart from household response rate

^e^Glasgow City Local Authority

Participation rates have fallen over the past decade in each country for which comparable data were available, apart from the Netherlands NLdeMaat Phase II (Table 5), with its substantial improvement in participation rates compared with phase I (Fig. 1).

**Table 5:** Changes in age-standardized national survey participation rates between 1998–2002 to 2010–2012 among 35–64 years old

Sex	Country^a^	Survey	Survey year		Blood pressure consent rate (%)		Average annual change in consent rate^b^	
			Year 1	Year 2	Year 1	Year 2	Absolute change pa^c^ (pp pa)	% change pa^d^ (% pa)
Men	Finland	FINRISK	2002	2012	62	55	−0.7	−1.1
	Germany	BGS98/DEGS	1998	2008-2011	61	49	−1.1	−1.9
	Netherlands	MORGEN / NLdeMaat Phase II	1993-1997^e^	2010^f^	40	39	−0.0	−0.1
	England^g^	HSE	2001	2011	70	55	−1.5	−2.1
Women	Finland	FINRISK	2002	2012	72	62	−1.0	−1.4
	Germany	BGS98/DEGS	1998	2008-2011	61	47	−1.3	−2.2
	Netherlands	MORGEN / NLdeMaat Phase II	1993-1997^e^	2010^f^	48	47	−0.1	−0.1
	England^g^	HSE	2001	2011	77	65	−1.2	−1.6

^a^Although each country studied had conducted at least two HESs, comparable data with the most recent survey were not available for the OEC (1998–2002) in Italy, SLAN 1997 in Ireland, and SHeS 1998 in Scotland; these countries have therefore been omitted from this table

^b^Where the survey was conducted over more than one year, the mean number of years from or to the midpoint of the survey was used

^c^Mean annual change in absolute response rate = (Rate for Year2 – Rate for Year1) / Number of years; pp pa: percentage points per annum

^d^Mean annual percentage change = (Rate for Year2 – Rate for Year1) *100 / (Rate for Year1 * Number of years)

^e^Age group 30–59; MORGEN-project, Amsterdam/Maastricht

^f^NLdeMaat phase 2: Age group 30–59, to permit comparison with results from MORGEN

^g^Among participants in co-operating households

**Fig. 2:**
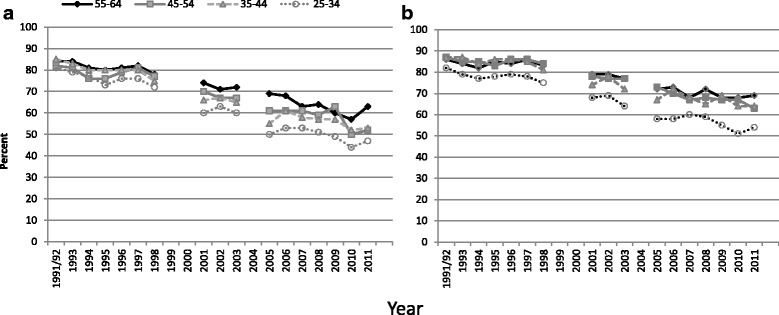
HSE consent rates for BP measurement, by age and sex, 1991/92 – 2011. Footnote to Fig. 2. **a** Men in co-operating households. **b** Women in co-operating households

**Fig. 3:**
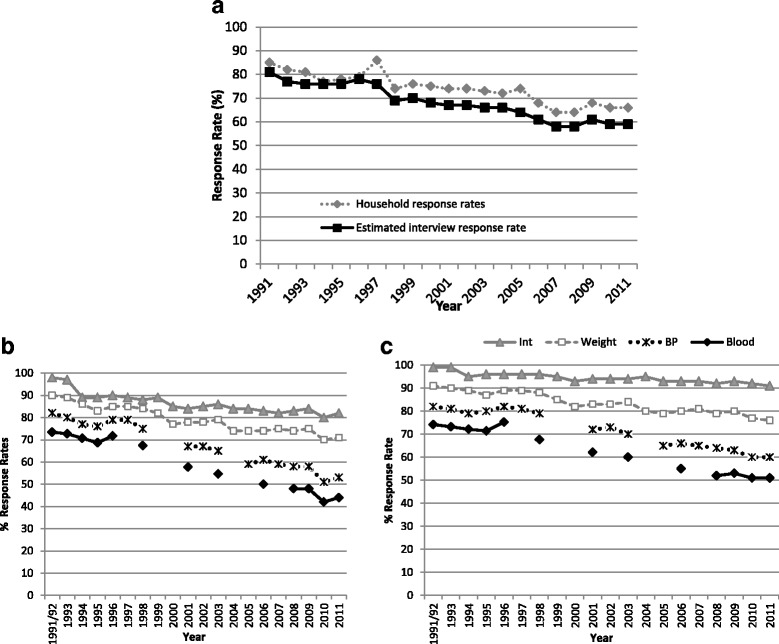
Participation rates by age and sex to different stages of the Health Survey for England, 1991–2011. Footnote to Fig. 3. **a** Household and estimated interview participation rates. **b** Men in co-operating households. **c** Women in co-operating households

**Fig. 4:**
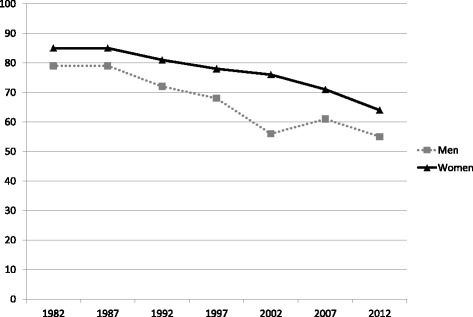
Changes in HES participation rates in Finland, by sex, 1982–2012. Footnote to Fig. 4: Data source: [70]

The average annual change in the other countries ranged from −0.7 to −1.5 pp; they were larger in women than men in Finland and Germany. In England, the falling participation rates for blood pressure measurement were greater in men than women and in younger than older people (Fig. 2). Falling participation rates in England occurred primarily for household participation (Fig. 3a), but even within co-operating households, participation in successive stages each fell over time to a greater extent (Fig. 3b and c). The reduction in initial participation rates over time in Finland (Fig. 4) is similar to the household and estimated interview participation rates in England over the same period.

Participation rates in the four countries using monetary incentives (England, Germany, the Netherlands and Scotland) were lower than in the other surveys. In Finland, which had the highest participation rate among individual-based surveys, pre-notification and a reminder before appointment time by short message service (SMS) was used. No clear correlation was observed between the range of examination days and hours of day offered and the participation rates obtained.

## Discussion

The main strength of this study is the inclusion of several European countries with significant experience of conducting nationally-representative health examination surveys. Differing definitions of response rates is a well-recognised issue [44, 45]. The limitation of these different definitions of non-response, in each individual survey, was overcome by use of agreed uniform definitions for both numerators and denominators. As with comparisons of the findings of disparate heath surveys across Europe, [46] the lack of uniformity of survey methods prevents assessment of the impact of cultural differences within countries, and aspects of sampling, recruitment, and data collection on participation rates. Analysis is therefore limited primarily to comparisons within each country.

In countries which have either national or local population registers, these were used as the sampling frames for individuals. Countries without population registers constructed their sample using address files. Population registers have good coverage of the population; updating procedures are usually regulated by law [47] and occur regularly. Population registers include information about the name of the person, their address and often their sex and age as well. Address files do not list individuals but buildings; they often include vacant buildings, offices and other non-domestic houses. The updating frequency of address files varies between countries. For example in Ireland, the GeoDirectory identified 2.7 % of residential addresses as vacant while the Census reported 15 % of residential addresses as vacant [41]. The large proportion of ineligible addresses in address files (>10 % on average) increases the cost of making contacts as interviewers have to check all addresses.

For practical reasons, people living in institutions are often excluded from health examination surveys, for example in the three surveys using household-based sampling. When people are asked to come to the fixed examination clinic, it may be impossible for institutionalized persons to participate. Also in cases where home visits are conducted routinely, or offered as an alternative for those not able to come to the examination site, inclusion of institutionalized persons may be difficult. Many institutionalized persons have illnesses which prevent their participation or ability to provide informed consent; in the case of prisoners, both the safety of the survey staff and the privacy of the examination may be difficult to guarantee. For the working aged population (18–64 years), the number of institutionalized people is a small proportion of the population and should not affect the representativeness of the survey results. In older age groups (65+ years), the proportion of the population living in institutions (such as care homes) is larger.3 %-6 % of people aged 64 and over in OECD countries, are living in long-term care institutions [48]: excluding them may affect the representativeness of the results.

The recent European recommendations (European Health Examination Survey) for conducting a national health examination survey determined that a sample size of at least 4000 persons is needed to survey the 25–64 year age group [49]. The EHES recommendations are similar to STEPS recommendations [50]. The sample size should be made up of equal numbers of men and women in each 10-year age group (500 in each group) in order to obtain nationally representative results. In each of the seven national health examination surveys, the sample size fulfilled this criterion even though in most of the surveys, a wider age group was covered.

Sending a short pre-notification letter for survey invitees before mailing out the invitation and questionnaire is technically easy to organize but for large population surveys it increases the cost of the survey. In some countries, sending a personalized pre-notification is not possible due to the sampling methods. In Finland, it was possible to send SMS messages for survey invitees as mobile phone numbers are widely available. In a recent, local HES within Finland, those receiving an SMS reminder were up to 25 pp more likely to participate [51], although telephone reminders have not been found to have a significant effect [25]. The rapid increase of mobile phone subscriptions and at the same time decline in land-line subscriptions is opening new possibilities for contacting survey invitees [52] in countries where listings of mobile phone numbers are available.

In health examination surveys, home visits have also been used to contact survey invitees who do not respond to the invitation [28]. In the Netherlands, this was an effective way to convince people to participate, although it was instituted at the same time as several other changes to the recruitment procedures, making formal evaluation difficult.

For health examination surveys, a Norwegian study reported that many participants felt that incentives would impose commercial features on the survey and could undermine the confidence in the survey. They also noted that there were clear differences between population groups regarding acceptance of incentives. People from lower socio-economic classes and younger age groups, the population groups which tend to have the lowest participation rates in health examination surveys, had more positive attitude towards incentives [27].

Although the larger the monetary incentive, the bigger the effect on participation rate [25], there are diminishing effects of greater amounts. In some countries, research ethics committees have prevented the use of monetary incentives in health examination surveys. Therefore, not all surveys have had an equal opportunity to use monetary incentives. In England, use of an unconditional £5 voucher in the advance letter to the household increased household participation rates by 3 % [53]. In large health examination surveys, providing monetary incentives can have a significant effect on the survey budget but it may also reduce number of contact attempts needed and therefore reduce survey staff costs.

Little has been reported about people’s preferences for day of the week and time of the day when it would be most convenient for them to participate in the survey. One would assume that availability depends on people’s daytime commitments and lifestage. It has been shown in the Health Survey for England that people attending the survey outside ‘office hours’ have different demographic and socio-economic profiles than those attending during the day on weekdays [29]. Therefore, it is important to organize health examination surveys so that everyone is able to be seen when convenient for the participant. This means offering a wide range of opening hours for examination clinics and/or home visits to maximize participation. On the other hand, extensive opening hours makes it difficult to organize collection of fasting blood samples (at least when 8–12 hours fasting is required), which are usually collected in mornings after overnight fasting. Longer data collection hours can also require more field work personnel, which increase the survey cost.

Health examination survey participation rates across seven European countries were generally higher in women and older people, whether conducted in participants’ own homes or an examination centre. Participation rates in the National Health and Nutrition Survey (NHANES) in the USA were also higher among women. Although participation rates in NHANES increased by age among women until recently, the declining participation with age among older men now also occurs in women [54].

Participation rates declined over time by 7–15 pp in the past decade in the four countries for which comparable data were available. In NHANES, reported (interview and) examination response rates fell from (99 % and) 74 % of adults aged 25-74y in 1971–74 to (70 % and) 67 % aged 30-69y^2^ in 2011-2012 [54]. These figures are not directly comparable with rates in our study as the NHANES data may not match our standardised definitions, however, the NHANES response rates remain high. It is possible that financial barriers to routine healthcare in the USA mean that the opportunity for a free health check has been a greater incentive in those countries than in European countries with universal healthcare.

Participation rates for England and Scotland presented in the Tables are based on households in which at least one adult responded, as the age and number of household members is otherwise unknown. After multiplying individual rates by household response rates, interview rates in England and Scotland were similar to or better than in other countries. However, the two-stage method used in England and Scotland for data collection had a marked reduction in participation in the second stage, with blood pressure measured in substantially fewer individuals than weight. There was further reduction in participation rates when blood sample consent was compared with blood pressure measurements, which occurred in the same survey nurse visit. This may reflect an unwillingness to provide blood samples in a domestic, non-clinical environment, particularly when the samples are not required for clinical reasons.

Participation rates in the examination elements in the five countries where participants attend a central location were uniformly high as a proportion of those interviewed, and participation rates for the three measurements varied little. However, the proportion attending as a percentage of the whole sample varied widely by age, sex, and country. In the Netherlands, the first phase of NLdeMaat had much lower participation rates, although already using more intensive recruitment than in the 1993–1997 MORGEN study. Phase II of NLdeMaat demonstrated that it was feasible to reproduce 'historical' participation rates, but at a much higher cost than previously.

The lower participation rate in capital cities has been noted before within individual studies [55]; we have confirmed this as a problem in most primate cities and metropolitan areas. Lower contact rates (a necessary first step before participation can be requested in surveys based on an interviewer calling in person, such as the household-based surveys) occur in more highly urbanized areas [56]. Groves and Couper relate this in part to these areas have a higher preponderance of less accessible household types, with more multi-unit dwellings, single-person households (thus a reduced likelihood of anyone being home when an interviewer calls), renter-occupied homes, more time spent commuting, and more entertainment options [56]. Large metropolitan areas also have a younger and more mobile population (increasing non-contact rates), greater poverty (also increasing non-contact [57]), and larger immigrant populations with more language barriers to survey participation, both through comprehension problems [33, 58] and language-related barriers of perceived relevance [58]. In the German DEGS1 study reported here, participation rates were inversely proportion to conurbation size (53 % in localities with a population <5,000; 35 % in cities of 500,000+) [59]. Participation rates were 10 pp higher among German than other nationals in DEGS1 [59].

The main concern over lower participation rates is the extent to which the results are representative of the underlying population, with non-response bias if participation is not random. Groves summarised five theoretical models relating response propensity to the likelihood of non-response bias [60]. He concluded that while non-response bias does occur, it is not a simple function of the non-response rate: higher response rates reduce the risk of such bias but not the actual presence of such bias. Non-respondents to health examination surveys have higher mortality than participants [11, 15–17]. Socio-economic position is associated with non-response [11, 23]; one study found that deprivation was associated with difficulty in making contact but individuals in less deprived areas had higher refusal rates [57]), Other studies found higher response rates among more educated individuals [13]. Some but not all studies have found both demographic and health differences between those responding early to a survey and those requiring greater effort to contact or to encourage into the survey [61, 62]. Representativity indicators have been proposed to replace response rates; they can be generated during fieldwork to target less represented potential participants (a responsive survey design) [63]. Field substitution to address differential non-response has little effect on the results [64] but post-survey adjustments (e.g. non-response; weighting) removes these differences, [61, 64, 65] with a suggestion to reduce fieldwork costs (or increase sample size) by reducing efforts to contact or convert non-responders and weighting data instead [61, 66].

Factors influencing decisions to participate in a survey include societal and individual level factors, survey design, and interviewer attributes [67]. In addition, the perceived relevance or sensitivity of the topic can affect participation. Thus health surveys may have health-related non-response bias both because those whose health is less good are more likely to be available [29] to contact, and because the survey may appear more salient to them. Gibson *et al.* have found reporting heterogeneity for similar health questions on limiting longterm illness, with lower prevalence among census respondents than health survey participants [68]. In contrast, alcohol consumption is often under-estimated by surveys; Gray et al. compared morbidity-linked survey and population data and proposed a method to develop more accurate (higher) alcohol consumption estimates using multiple imputation [69]. However, Hall et al. found that non-response weighting bias addressed health variables adequately, other than smoking, but not attitudinal variables [66].

## Conclusion

Seven national health examination surveys conducted in Europe in 2007–2012 can be classified either as individual-based or as household-based surveys. For each survey, the best available sampling frame within the country was used and recruitment methods were adjusted for national needs. Participation rates differ by age, sex, country, and survey design but have fallen over time in most countries. There is no single correct way to organize a national HES but national circumstances, cultural requirements and norms, and the resources available have important roles in effective survey organization. Experience in the Netherlands shows that higher participation rates can be maintained through intensive efforts but these incur considerable costs.

## Footnotes

1 The primate city is a major city that is at least twice as large as the next largest city and more than twice as significant, e.g. Greater Glasgow in Scotland.

2 Data are not published for the same age groups in earlier and more recent years. In 2011–12, response rates were substantially higher among those aged 30-69y than those 20-29y or 70+, so the age-groups presented in the text underestimate the fall in response rates.

## References

[1] Grotvedt L, Kuulasmaa K, Tolonen H, Heldal J, Graff-Iversen S, Tolonen H, Koponen P, Aromaa A, Conti S, Graff-Iversen S, Grotvedt L, Kanieff M, Mindell J, Natunen S, Primatesta P (2008). 7. Sampling and recruitment. Review of Health Examination Surveys in Europe.

[2] STEPwise approach to surveillance (STEPS) http://www.who.int/chp/steps/en/ (Accessed 07/09/2015)

[3] Borodulin K, Vartiainen E, Peltonen M, et al. Fourty-year trends in cardiovascular risk factors in Finland. Eur J Public Health. 2014. 10.1093/eurpub/cku174 25422363

[4] Oyebode O, Mindell J (2013). Use of data from the Health Survey for England in obesity policy making and monitoring. Obesity Reviews.

[5] Tolonen H, Koponen P, Mindell JS, Mannisto S, Giampaoli S, Dias CM, et al. Under-estimation of obesity, hypertension and high cholesterol by self-reported data: Comparison of self-reported information and objective measures from health examination surveys. Eur J Public Health. 2014, doi:10.1093/eurpub/cku074. 10.1093/eurpub/cku074 24906846

[6] Tolonen H, Koponen P, Mindell J, Mannisto S, Kuulasmaa K (2014). European Health Examination Survey--towards a sustainable monitoring system. Eur J Public Health.

[7] Biemer PP (2004). Measurement errors in surveys.

[8] Blackman S, Goldstein KM (1968). Some aspects of a theory of community mental health. Community Ment Health J.

[9] Boshuizen HC, Viet AL, Picavet HS, Botterweck A, van Loon AJ (2006). Non-response in a survey of cardiovascular risk factors in the Dutch population: determinants and resulting biases. Public Health.

[10] Drivsholm T, Eplov LF, Davidsen M, Jorgensen T, Ibsen H, Hollnagel H (2006). Representativeness in population-based studies: a detailed description of non-response in a Danish cohort study. Scand J Public Health.

[11] Harald K, Salomaa V, Jousilahti P, Koskinen S, Vartiainen E (2007). Non-participation and mortality in different socioeconomic groups: the FINRISK population surveys in 1972–92. J Epidemiol Community Health.

[12] Sogaard AJ, Selmer R, Bjertness E, Thelle D (2004). The Oslo Health Study: The impact of self-selection in a large, population-based survey. Int J Equity Health.

[13] Tolonen H, Dobson A, Kulathinal S (2005). Effect on trend estimates of the difference between survey respondents and non-respondents: results from 27 populations in the WHO MONICA Project. Eur J Epidemiol.

[14] Van Loon AJ, Tijhuis M, Picavet HS, Surtees PG, Ormel J (2003). Survey non-response in the Netherlands: effects on prevalence estimates and associations. Ann Epidemiol.

[15] Hara M, Sasaki S, Sobue T, Yamamoto S, Tsugane S (2002). Comparison of cause-specific mortality between respondents and nonrespondents in a population-based prospective study: ten-year follow-up of JPHC Study Cohort I. Japan Public Health Center. J Clin Epidemiol.

[16] Jousilahti P, Salomaa V, Kuulasmaa K, Niemela M, Vartiainen E (2005). Total and cause specific mortality among participants and non-participants of population based health surveys: a comprehensive follow up of 54 372 Finnish men and women. J Epidemiol Community Health.

[17] Une H, Miyazaki M, Momose Y (2000). Comparison of mortality between respondents and non-respondents in a mail survey. J Epidemiol.

[18] Boniface DRE, Tefft ME (2002). Dietary fats and 16-year coronary heart disease mortality in a cohort of men and women in Great Britain. Eur J Clin Nutr.

[19] Atrostic BK, Bates N, Burt G, Silberstein A (2001). Nonresponse in U.S. government household surveys: consistent measures, recent trends, and new insights. J Off Stat.

[20] De Heer W (1999). International reponse trends. Results of an international survey. J Off Stat.

[21] de Leeuw E, de Heer W, Groves R, Dillman D, Eltinge J, Little RJ (2002). Trends in Household Survey Nonresponse: A Longitudinal and International Comparison. Survey Nonresponse.

[22] Aromaa A, Koponen P, Tafforeau J, Vermneire C, Primatesta P, Marmot M (2003). Health surveys: evaluation and recommendations. Subproject reports of Phase 2 of the project Health surveys in the EU: HIS and HIS/HES Evaluations and Models.

[23] Tolonen H, Helakorpi S, Talala K, Helasoja V, Martelin T, Prattala R (2006). 25-year trends and socio-demographic differences in response rates: Finnish adult health behaviour survey. Eur J Epidemiol.

[24] Williams B, Entwistle V, Haddow G, Wells M (2008). Promoting research participation: Why not advertise altruism?. Soc Sci Med.

[25] Edwards PJ, Roberts I, Clarke MJ, Diguiseppi C, Wentz R, Kwan I (2009). Methods to increase response to postal and electronic questionnaires. Cochrane Database Syst Rev.

[26] Church AH (1993). Estimating the effect of incentives on mail survey response rates: A meta-analysis. Public Opin Quartely.

[27] Antonsen S (2005). Motivasjon for deltakelse i helseundersokelser. Norsk Epidemiologi.

[28] Holle R, Hochadel M, Reitmeir P, Meisinger C, Wichmann HE (2006). Prolonged recruitment efforts in health surveys: effects on response, costs, and potential bias. Epidemiology.

[29] Mindell J, Aresu M, Becares L, Tolonen H (2012). Representativeness of participants in a cross-sectional health survey by time of day and day of week of data collection. Eur J Public Health.

[30] Galea S, Tracy M (2007). Participation rates in epidemiologic studies. Ann Epidemiol.

[31] Tunstall-Pedoe H, Kuulasmaa K, Tolonen H, Davidson M, Mendis S, for The WHOMP (2003). MONICA Monograph and Multimedia Sourcebook.

[32] Gosswald A, Lange M, Dolle R, Holling H (2013). The first wave of the German Health Interview and Examination Survey for Adults (DEGS1): participant recruitment, fieldwork, and quality management. Bundesgesundheitsblatt Gesundheitsforschung Gesundheitsschutz.

[33] Kamtsiuris P, Lange M, Hoffmann R, Schaffrath Rosario A, Dahm S, Kuhnert R (2013). The first wave of the German Health Interview and Examination Survey for Adults (DEGS1): sample design, response, weighting and representativeness. Bundesgesundheitsblatt Gesundheitsforschung Gesundheitsschutz.

[34] Giampaoli S, Vanuzzo D, Survey edGdRdPOECHE (2014). La salute cardiovascolare degli italiani. G Ital Cardiol.

[35] Nederland de Maat Genomen http://www.rivm.nl/Onderwerpen/Onderwerpen/N/Nederland_de_Maat_Genomen (Accessed 07/09/2015)

[36] Blokstra A, Vissink P, Venmans LAMJ, Holleman P, van der Schouw YT, Smit HA (2011). Nederland de Maat Genomen, 2009–2010. Monitorin van risicofactoren in de algemene bevolking. RIVM Rapport 260152001/2011.

[37] Craig R, Mindell J (2012). Health Survey for England 2011. Volume 2. Methods and documentation.

[38] Mindell J, Biddulph JP, Hirani V, Stamatakis E, Craig R, Nunn S (2012). Cohort Profile: The Health Survey for England. Int J Epidemiol.

[39] Gray L, Batty GD, Craig P, Stewart C, Whyte B, Finlayson A (2010). Cohort Profile: The Scottish Health Surveys Cohort: linkage of study participants to routinely collected records for mortality, hospital discharge, cancer and offspring birth characteristics in three nationwide studies. Int J Epidemiol.

[40] Rutherford L, Sharp C, Bromley C (2012). The Scottish Health Survey 2011. Volume 3: Technical Report.

[41] Morgan K, McGee H, Watson D, Perry I, Barry M, Shelley E (2008). SLÁN 2007: Survey of Lifestyle, Attitudes & Nutrition in Ireland. Main Report.

[42] Jefferson M (1939). The law of the primate city. Geogr Rev.

[43] Ahmad OB, Boschi-Pinto C, Lopez AD, Murray CJL, Lozano R, Inoue M, Organization WH (2001). Age standardization of rates: a new WHO standard. GPE Discussion Paper Series: No 31 EIPH/GPE/EBD.

[44] Wiseman F, Billington M (1984). Comment on a Standard Definition of Response Rates. J Mark Res.

[45] Lynn P, Beerten R, Laiho J, Martin J (2001). Recommended Standard Final Outcome Categories and Standard Definitions of Response Rate for Social Surveys. Working Papers of the Institute for Social and Economic Research.

[46] Gray L, Merlo J, Mindell J, Hallqvist J, Tafforeau J, O’Reilly D (2012). International differences in self-reported health measures in 33 major metropolitan areas in Europe. Eur J Public Health.

[47] Tolonen H (2005). Towards the high quality of population health surveys. Standardization and quality control.

[48] OECD. Society at a Glance 2006. OECD Social Indicators. In.: OECD Publishing. doi: 10.1787/soc_glance-2006-en; 2007.

[49] Heldal J, Jentoft S. 2. Target population and sample size. In: Helsinki TH, editors. EHES Manual Part A Planning and preparation of the survey. Volume Directions 2013_001. National Institute for Health and Welfare; 2013. Available at http://urn.fi/URN:ISBN:978-952-245-842-1. (Accessed 07/09/2015)

[50] World Health Organization (2014). STEPS Manual.

[51] Tolonen H, Aistrich A, Borodulin K (2014). Increasing health examination survey participation rates by SMS reminders and flexible examination times. Scand J Public Health.

[52] World Telecommunication/ICT Indicators database 2013 (17th Edition) [http://www.itu.int/en/ITU-D/Statistics/Pages/publications/wtid.aspx]. (Accessed 07/09/2015).

[53] Craig R, Mindell J, Hirani V (2009). Health Survey for England 2008. Volume 2. Methods and documentation.

[54] NHANES response rates [www.cdc.gov/nchs/nhanes/response_rates_cps.htm]. (Accessed 07/09/2015).

[55] Mindell JS, Tipping S, Pickering K, Hope S, Roth MA, Erens B (2010). The effect of survey method on survey participation: Analysis of data from the Health Survey for England 2006 and the Boost Survey for London. BMC Med Res Methodol.

[56] Groves RM, Couper MP (1998). Nonresponse in household interview surveys.

[57] Goodman A, Gatward R (2008). Who are we missing? Area deprivation and survey participation. Eur J Epidemiol.

[58] Ahlmark N, Algren M, Holmberg T, Norredam M, Nielsen S, Blom A (2015). Survey nonresponse among ethnic minorities in a national health survey - a mixed-method study of participation, barriers, and potentials. Ethn Health.

[59] Pelzl I, Pohlabeln H, Relneke A, Ahrens W (2013). Externe Qualitatssicherung der ersten Welte der Studie zur Gesundheit Erwachsener in Deutschland (DEGS1). [External quality assuranceof the first wave of the German Health Interview and Examination Survey for Adults (DEGS1)]. Bundesgesund Heitsbl.

[60] Groves RM (2006). Nonresponse rates and nonresponse bias in household surveys. Public Opin Quartely.

[61] Davern M, McAlpine D, Beebe TJ, Ziegenfuss J, Rockwood T, Call KT (2010). Are Lower Response Rates Hazardous to Your Health Survey? An Analysis of Three State Telephone Health Surveys. Health Serv Res.

[62] Korkeila K, Suominen S, Ahvenainen J, Ojanlatva A, Rautava P, Helenius H (2001). Non-response and related factors in a nation-wide health survey. Eur J Epidemiol.

[63] Shlomo N, Skinner C, Schouten B (2012). Estimation of an indicator of the representativeness of survey response. J Stat Planning Infer.

[64] Van der Heyden J, Demarest S, Van Herck K, De Bacquer D, Tafforeau J, Van Oyen H (2014). Association between variables used in the field substitution and post-stratification adjustment in the Belgian health interview survey and non-response. Int J Public Health.

[65] Forthofer RN (1983). Investigation of nonresponse bias in NHANES II. Am J Epidemiol.

[66] Hall J, Brown V, Nicolaas G, Lynn P (2013). Extended field efforts to reduce the risk of non-response bias: Have the effects changed over time? Can weighting achieve the same effects?. Bull Méthode Social.

[67] Groves RM, Cialdini RB, Couper MP (1992). Understanding the decision to participate in a survey. Public Opin Q.

[68] Gibson A, Hewson P, Asthana S (2013). Modelling the nature, scale and consequence of health-related non-response bias in Health Survey for England data. Health Survey Users Meeting.

[69] Gray L, McCartney G, White IR (2013). Use of record-linkage to handle non-response and improve alcohol consumption estimates in health survey data: a study protocol. BMJ Open.

[70] Vartiainen E, Borodulin K, Sundvall J, Laatikainen T, Peltonen M, Harald K (2012). FINRISKI Tutkimus: Väestön kolesterolitaso on vuosikymmenien laskun jälkeen kääntynyt nousuun. Suomen Lääkärilehti (Finnish Medical Journal).

